# The nation‐state, class, digital divides and social anthropology

**DOI:** 10.1111/1469-8676.12830

**Published:** 2020-05-15

**Authors:** David N. Gellner

**Affiliations:** ^1^ School of Anthropology and Museum Ethnography University of Oxford Oxford OX2 6PE UK

The events of 2020 are a powerful demonstration that the decline of the nation‐state in the age of hyper‐globalisation or ‘overheating’ has, like Mark Twain’s death, been greatly exaggerated. Throughout the world (with interesting local contrasts in North America, East Asia, Scandinavia and South Asia) a massive cross‐national social‐science experiment is being carried out in real time as different strategies are adopted by neighbouring countries. It feels as if, for better or worse, we are living through a radical turning point. In the face of an existential threat, the old gods of neoliberalism are being thrown on a bonfire.

It is an ethnographic truism that, in moments of crisis, underlying power structures and unspoken truths are revealed. The current unprecedented challenge to globalisation and capitalism is no exception. Political journalists, adept at ‘deep hanging out’ with the holders of power, are stunned at the way advocates of shrinking the state are now pumping previously unimaginable quantities of money into the economy: contrary to what Theresa May once declared, there is a magic money tree after all. In the UK £18 billion of accumulated NHS (National Health Service) debt has been written off at a stroke following years of cuts and forced marketisation that played a huge role in producing the chaos visible in the British response to COVID‐19.

The class divide in lockdown living is evident in the UK, but nowhere is it more extreme than in South Asia. The mis‐named ‘social distancing’ is a cruel joke when people live eight or ten to a room and when a dozen households may share the same water tap and a couple of latrines. In India, lockdown was imposed with four hours’ notice, without planning or forethought for what would happen to the poor whose means of livelihood were lost overnight. Hundreds of thousands set off to walk to their home villages in scenes reminiscent of the Partition exodus in 1947, minus pervasive communal massacres, but with random and callous police harassment. Hundreds of Nepalis trying to go home from India remain blocked at border crossings while their government dithers over setting up quarantine camps. In India, hate‐filled social media posts blaming Muslims for the virus circulated on WhatsApp within hours of Modi’s characteristically abrupt and authoritarian decision.

At the global level, we need to listen to specialists in medical and disaster anthropology. One of the prime lessons of the latter field is that ‘there is no such thing as a natural disaster’. Social and political arrangements determine who suffers and how. Politicians have insisted: ‘We are following the science’. But, in the face of radical uncertainty, scientists don’t agree and there are many types of science. Learning to live with complexity and to plan for, and act on, ‘wicked problems’ might, one hopes, be one of many political lessons learned.

Theorists of the networked society are seeing their analyses become ever more accurate by the day. We social anthropologists face an existential threat to our method. We must all learn to do research online. Activities that previously felt like guilty procrastination can now be relabelled netnography, but this leaves the digital divide, between those online and those barely online or not online at all, more gaping than ever. As the lockdown recedes, it will be imperative for social anthropologists to get out and about to make contact with the digitally disenfranchised.

